# Coexistence of Factor VII Deficiency and Hereditary Spastic Paraplegia in Two Siblings

**DOI:** 10.1155/2016/1351873

**Published:** 2016-11-27

**Authors:** Hortensia De la Corte-Rodriguez, E. Carlos Rodriguez-Merchan, M. Teresa Alvarez-Roman, Ana L. Hernandez-Moreno

**Affiliations:** ^1^Department of Physical Medicine and Rehabilitation, La Paz University Hospital, Madrid, Spain; ^2^Department of Orthopaedic Surgery, La Paz University Hospital, Madrid, Spain; ^3^Department of Haematology, La Paz University Hospital, Madrid, Spain

## Abstract

We present the case of two patients aged 12 years and 7 years who were referred to our hospital for factor VII deficiency inherited in an autosomal recessive pattern, who had suffered from previous multiple joint haemarthroses. They presented with fine motor symptoms and difficulty in walking. During physical examination we observed neurological symptoms (general hypotonia, muscular hypotrophy, exaggerated tendon reflexes, pes cavus, and spastic gait). Given that the symptoms were not justified by the deficiency of coagulation factor VII and on suspicion of hereditary spastic paraplegia (HSP), tests were carried out. Findings from the tests confirmed the diagnosis of HSP (axonal degeneration of the central motor pathway and pyramidal tracts), further complicated by mixed neuropathy. This disease was also inherited in an autosomal recessive pattern with no direct genetic association with factor VII deficiency. Neurological symptoms had gone unnoticed due to a history of multiple joint haemarthrosis; musculoskeletal examination led to a satisfactory differential diagnosis. Haematological prophylaxis was commenced with rFVIIa at 30 mcg/kg, three days per week. A rehabilitation programme was prescribed so that the patient could remain independent for as long as possible, based on orthosis, physiotherapy, and occupational therapy. Response to treatment is currently satisfactory and no new bleeding has presented. As far as we are aware, the coexistence of these two diseases (factor VII deficiency and HSP) has not been previously reported in the literature.

## 1. Introduction

Factor VII (FVII) deficiency is a rare blood clotting condition, with an autosomal recessive inheritance pattern, and may affect both men and women [1]. There is little correlation between the blood plasma levels and bleeding signs. However, it is accepted that levels exceeding 25% are safe, even for surgical interventions [2]. The most frequent clinical presentation is skin or mucosal tissue bleeding (menorrhagias, epistaxis, and postdental extraction bleeding) and, on rare occasions, haemarthrosis. Mild and moderate types occur most frequently and make up two-thirds of the total, with serious haemorrhages representing approximately 5% of bleeding [3]. Therapeutic options vary within the use of recombinant FVII, prothrombin complex concentrates (PCCs), or fresh frozen plasma (FFP) [2]. FVII deficiency is rarely associated with other genetic disorders.

Hereditary spastic paraplegia (HSP) is a hereditary disease inherited in an autosomal dominant or autosomal recessive pattern or as a chromosome X-linked condition [4]. Symptoms involve the central motor pathway, originating in the neurons and pyramidal tracts (axonal degeneration), producing “pure” forms (with spastic paraparesis which may be accompanied by urinary bladder urgency and hypoesthesia) and “complicated” forms (when spastic paraparesis is accompanied by other symptoms and signs such as oligophrenia, ataxia, neuropathy, deafness, cataracts, or amyotrophy) [5]. There is no aetiological treatment for this disease and therapy is therefore used to treat symptoms (of paresis, spasticity, pes cavus, urinary disorders, etc.).

We present what appears to be the first published case of two siblings with hereditary autosomal recessive FVII deficiency coexisting with HSP.

## 2. Case Report

The older brother is a patient aged 12 who has recently been transferred to our hospital and who was diagnosed with severe FVII deficiency aged 11 months in his country of origin. At 8 years of age he started intermittent preventative treatment with 1.5 mg recombinant factor VII, which was disrupted due to shortages (he had not received preventative therapy for over a year). Family history showed that his parents are first cousins (the parents of both parents were siblings) and “carriers” of the FVII deficiency.

He mentioned that he had suffered from spontaneous bruising and multiple haemarthroses on his elbows, wrists, hips, knees, and ankles (>10 during the last year), with no suffering from haemorrhages in other organs. Findings from tests carried out in our laboratory showed a normal haemogram and a 6.9% FVII. He was not in pain but did have difficulty walking and doing fine motor movements. He was able to eat and dress independently but required assistance. He did not do any physical activity. He could walk without sticks but with a foot orthosis prescribed in his country of origin.

**Figure 1:**
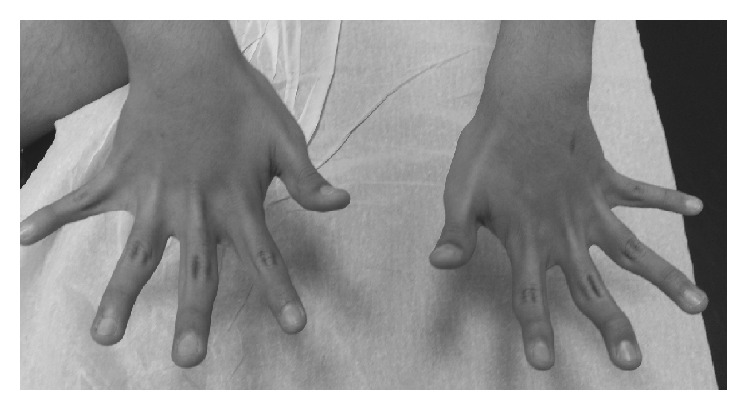
Clinical view of the 12-year-old patient showing marked hypotrophy of the intrinsic muscles groups of his hands and fingers.

**Figure 2:**
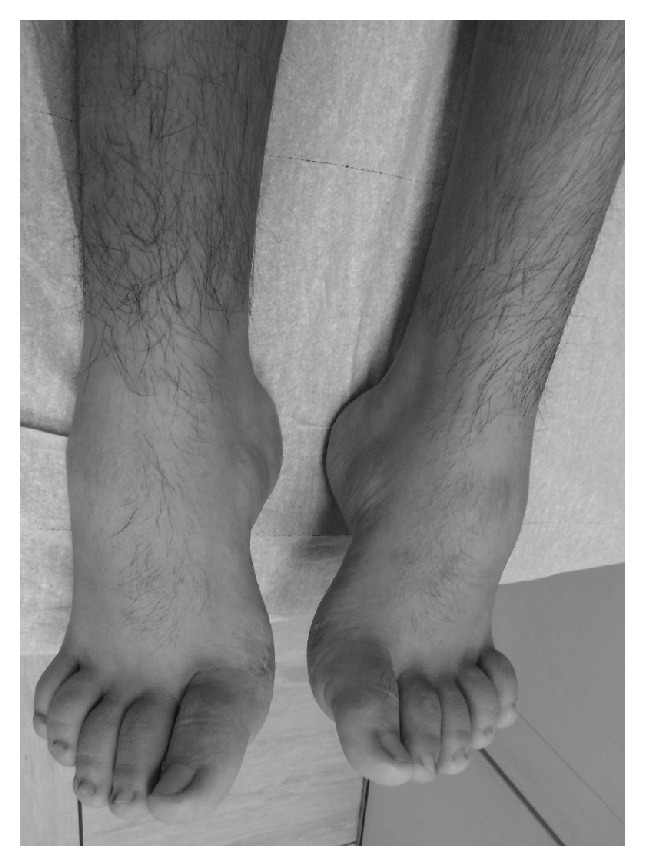
The feet of the 12-year-old patient show pes cavus and equinovarus deformity caused by the existing muscular imbalance.

Musculoskeletal examination showed deformity in his right elbow with joint mobility restriction (flexion of 115°, extension of −15°) and periarticular muscular hypotrophy related to posthaemarthrosis arthropathy. The other joints were functioning and had a Haemophilia Joint Health Score (HJHS) of 10. Neurological examination showed normal cognition, general hypotonia, strength maintained, exaggerated tendon reflexes, positive Babinski reflex, pes cavus, and more distally marked muscular hypotrophy (Figures 1 and 2). Steppage gait with varus support showed that he was not able to jump or stand on one foot; he went upstairs with support and without alternating both feet. Given that the symptoms were not justified by his factor VII deficiency and on suspicion of HSP, his parents and sister were examined.

His 7-year-old sister, with serious FVII deficiency (this tested as 8.6% in our laboratory), presented with arthropathy of the right knee (HJHS score of 3) and fewer neurological symptoms: general hypotonia and hyperreflexia, with a tendency to pes cavus and difficulty in ascending stairs without help.

The following tests were carried out: visual and auditory evoked potentials (normal latencies), somatosensory evoked potentials (with no spinal cord or cerebral responses), genetic testing, MRI (magnetic resonance imaging), and EMG (electromyogram). Results confirmed the diagnosis of spastic paraplegia complicated with axonal motor polyneuropathy in both cases.

**Figure 3:**
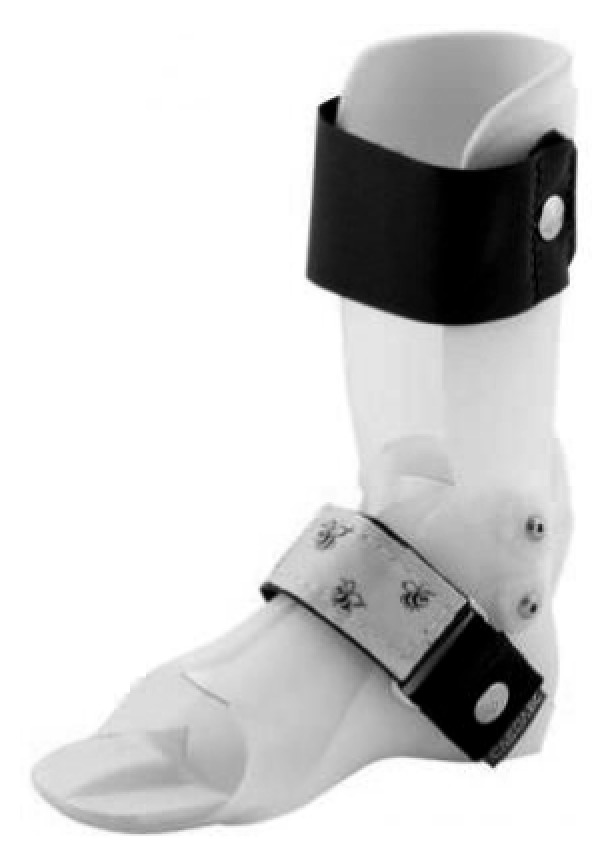
Dynamic ankle foot orthosis prescribed to the 12 year old patient to provide him with good support of the foot and ankle when walking.

Following primary assessment prophylaxis was initiated with rFVIIa at 30 mcg/kg, a total 1.5 mg dose (older brother), and 0.5 mg dose (younger sister), three days per week (Monday-Wednesday-Friday). A rehabilitation programme was prescribed which aimed at maintaining functional independence for the longest possible time, based on physiotherapy (inhibitory techniques of spasticity, polyarticular assisted kinesiotherapy, progressive muscular strengthening, proprioception, and balance and coordination) and occupational therapy (fist and grip strength, bimanual dexterity, gestural readaptation, reeducation of activities of daily living, and compensatory manoeuvres). It was also necessary to prescribe a DAFO (dynamic ankle foot orthosis) to the older brother to control his pes cavus-varus (Figure 3). Response to treatment is satisfactory at present and there has been no further presentation of bleeding. Both patients are currently being monitored by the haematology, rehabilitation, and neuropaediatric departments.

## 3. Discussion

In a patient with factor VII deficiency and a background of previous multiple joint haemarthrosis, walking disorders from knee and ankle arthropathy are quite common. The reported symptoms are pain, deformity, a lack of joint mobility, muscular hypotrophy which are associated with symptoms of arthropathy derived from the haemarthrosis [6]. It is important to recall that amyotrophies, muscular imbalances, and weakness are also neurological symptoms, with which we may rule out other diseases of neuromuscular origin. For all these reasons, presenting those cases, we would like to emphasise the importance of having an exhaustive musculoskeletal examination, for a satisfactory differential diagnosis of the problems derived from coagulopathy and neurological disease [6].

It has been reported that both FVII deficiency and HSP may have an autosomal recessive inheritance pattern [2, 4], as is the case of the siblings we present here. FVII is one of the vitamin K-dependent plasma glycoproteins and is coded in chromosome 13 (a broad spectrum of mutations has been characterised in the FVII gene) [1, 2]. HSP is genetically heterogeneous (with 32 loci described up to now) characterised by debility and spasticity in lower limbs with pyramidal tracts in the “pure” forms and other signs in the “complicated” forms, as in the cases we present which involved peripheral neuropathy; spastic gait is the most characteristic symptom. Symptoms may occur at any age, ranging between the first and seventh decades of life [4, 5]. In the older brother neurological symptoms were more apparent, whilst his sister was progressively developing them. Up to now, we have found no direct genetic link between both diseases. The existing familial relationship between the parents (first cousins) is of note. They were unaware of their condition as carriers and transmitted both diseases to their children.

To conclude, we present what we believe to be the first published case of factor VII deficiency complicated with coexisting HSP, with an autosomal recessive inheritance pattern, in two siblings aged 12 years and 7 years. The neurological disease had not been apparent because functional deficiencies had initially been considered with multijoint haemarthroses secondary to factor VII deficiency. Thorough musculoskeletal and neurological examination led to clinical suspicion, which was essential for concluding an early diagnosis of complicated HSP and the application of an adequate haematological and rehabilitating treatment appropriate for the current situation and the expected development of combined diseases. Our final aim was to maintain the greatest possible autonomy when walking and performing everyday activities to improve our patients' quality of life.
